# Effect of Organic Manures on Growth, Yield, Leaf Nutrient Uptake and Soil Properties of Kiwifruit (*Actinidia deliciosa* Chev.) cv. Allison

**DOI:** 10.3390/plants11233354

**Published:** 2022-12-02

**Authors:** Sunny Sharma, Vishal Singh Rana, Neerja Rana, Umesh Sharma, Kasahun Gudeta, Khadiga Alharbi, Fuad Ameen, Sartaj Ahmad Bhat

**Affiliations:** 1 Department of Fruit Science, College of Horticulture, Dr. Yashwant Singh Parmar University of Horticulture and Forestry, Solan 173230, India; 2 Department of Basic Sciences, College of Forestry, Dr. Yashwant Singh Parmar University of Horticulture and Forestry, Solan 173230, India; 3 Department of Tree Improvement and Genetic Resources, College of Forestry, Dr. Yashwant Singh Parmar University of Horticulture and Forestry, Solan 173230, India; 4 School of Biological and Environmental Sciences, Shoolini University of Biotechnology and Management Sciences, Solan 173229, India; 5 Department of Applied Biology, Adama Science and Technology University, Adama 1888, Ethiopia; 6 Department of Biology, College of Science, Princess Nourah bint Abdulrahman University, Riyadh 11671, Saudi Arabia; 7 Department of Botany and Microbiology, College of Science, King Saud University, Riyadh 11451, Saudi Arabia; 8 River Basin Research Center, Gifu University, Gifu 501-1193, Japan

**Keywords:** *Actinidia deliciosa*, nutrient uptake, organic manure, yield, spad values, physicochemical properties

## Abstract

In recent decades, organic kiwifruit farming has come up as a feasible method for high-quality kiwi production without using chemical fertilizers. The primary objective of this research was to investigate how the sole application of organic and the combined application of organic manures affected the growth, yields, and quality of Allison kiwifruit, as well as the soil’s physicochemical characteristics. The field trial was conducted on cv. Allison to determine the efficacy of organic manures (OM) on growth, nutrient absorption, production and soil health. The experiment involved eight treatments, viz.: T_1_: 100% Dairy manure (DM); T_2_: 100% Vermicompost (VC); T_3_: 100% chicken manure (CM); T_4_: 50% DM + 50% CM; T_5_: 50% DM + 50% VC; T_6_: 50% CM + 50% VC; T_7_: DM + CM + VC in equal proportions; and T_8_: Recommended nutrients inorganic NPK + 40 kg DM. A randomized complete block design comprising three replicas was used in this investigation. The use of inorganic fertilizers (NPK) in combination with DM enhanced Spad Values Chlorophyll, fruit production, leaf number, leaf area, and stem diameter while also improving the soil’s chemical characteristics. The flower initiation was recorded with DM and Vermicompost (50:50). Furthermore, when compared to inorganic fertilizer treatment, OM treatment significantly improved fruit quality by improving fruit chemical composition in terms of soluble solids contents and leaf nutrient status, as well as improving soil’s physical properties with DM and Vermicompost (50:50). The study’s outcome revealed that OM had a significant impact on flowering time, fruit SSC, leaf nutritional status, and soil physical characteristics. In comparison to organic treatments, recommended fertilizer dosages (NPK + DM) improved plant growth, fruit yield, and soil chemical characteristics.

## 1. Introduction

After China, India is the world’s second-largest fruit grower [1]. In recent decades, drastic changes in fruit production under the mid-hills zone of the Himalayan climate have been depicted [2]. Therefore, introducing new exotic varieties or developing new cultivars such as low-chill peach and kiwifruit has extended their scope for cultivation in the mid-hills under changing climatic scenarios [3]. Several fruit crops have come to prominence in the western Himalayan regions during the last few decades. One of these is the kiwifruit, which is also known as the Chinese gooseberry [4]. Botanically, kiwifruit is a deciduous vine that originated in southern China [5]. Kiwi is recognized across the world for its distinct flavor, which includes sweet and slightly acidic flesh, high nutritional value minerals and beneficial metabolites, particularly the high ascorbic acid vitamin-C content [6].

Chemical fertilizers, organic manures (OM), and their combinations have shown significant effects on sustainable food production and are an essential constituent of various agricultural systems [7]. Modern agricultural systems rely heavily on synthetic fertilizers and agrochemicals [8]. Fertilizer is one of the most important inputs, accounting for approximately a third of the total cost of cultivation [9]. The fast depletion of nitrogenous, phosphatic, and potassic reserves because of extreme usage is a cause of grave concern for countries that are yet to achieve food security [10]. As per the guidelines of the FAO for good agricultural practices (GAP), the use of agri-inputs such as agrochemicals should be avoided or used to a limited extent for cultivation [11]. Furthermore, the residues of pesticides and heavy metals in crops are a major global issue in the marketing of commodities [12]. 

Continuous and indiscriminate application of chemical fertilizers has degraded the soil’s health in terms of fertility. The low productivity in horticultural output has resulted from a decline in soil fertility. Organic manures, bio-fertilizers, and liquid manures are increasingly being used as alternatives to chemical fertilizers because of growing fertilizer costs and their negative effects on soil health [13]. In recent years, organic fruit production technology has gained momentum, both in terms of consumer demand and as a genuine desire of many fruit orchardists to sustain crop production and soil health [14]. Organic produce fetches a higher price, which has prompted producers to grow fruit crops organically [3]. Furthermore, organic nutrient management has a significant impact on the soil’s physico-chemical characteristics and results in healthier output [15]. 

In these circumstances, organic cultivation of kiwifruit may be a promising technology to produce quality fruit since it provides a healthier substitute to cultivation involving excessive use of chemical fertilizers [16]. Unfortunately, only a few attempts have been made to determine the impact of organic manures and their combinations on the development, production, quality, and nutritional status of kiwifruit cv. ‘Allison’ [17]. The demand for products in organic agriculture, with special reference to fruit products, has shown an increasing trend in recent years. The increasing cost of fertilizers, growing ecological concerns and conservation of energy have created considerable interest in the use of organics as a source of plant nutrients. Organic farming is a production system that avoids or largely excludes the use of synthetically compounded fertilizers, growth regulators and livestock feed additives [2,3]. The concept of organic farming has created a renewed interest in the use of organic manures. This intensive use of agrochemicals has reduced biodiversity, increased irreversible soil erosion and reduced soil organic matter [15]. These organic approaches are more economical, renewable, and eco-friendly. The increasing cost of fertilizers and global concern of groundwater pollution through leaching from the soils are discounting the use of fertilizers [16]. To overcome these problems, there is widespread interest in developing sustainable agricultural systems which exclude external inputs, especially fertilizers and herbicides, minimizing the impacts on the environment and soils [17]. The use of synthetic pesticides slows down the defense mechanisms against pathogens with the consequence of favoring primary metabolism. The cultural practices may result in different plant composition and nutritional quality, which in turn influence the cold storage performance of the products. These differences in fertility and pest management, affect the allocation of secondary plant metabolites such as ascorbic acid and phenolic compounds. The present challenge of feeding the world requires a new strategy to ensure food security which is surely based on food availability and access, but also food safety and nutritional quality. Organic production systems may be a way to ensure the sustainability of production, allowing the preservation of natural resources for present and future generations [16]. The relevant literature on the use of different organic formulations, namely, DM, vermicompost, Poultry Manure, Neem cake, Jeevamrit and Panchgavya about plant growth, yield, fruit quality and soil characteristics. Organic manures along with inorganic fertilizers would have given micronutrients at an optimal level because of their higher effectiveness. Presently, many brands of commercial organic manures are available on the market, but the suitability of these manures for kiwifruit production is less well-known. Keeping in view the positive effects of organic manures on the growth, yield, and quality of fruits, the proposed investigation was undertaken to check the differential responses of organic manures on the growth, yield, quality of kiwifruit and the soil properties of the orchard.

## 2. Results

### 2.1. Chlorophyll Content and Flowering Characteristics

**Figure 1:**
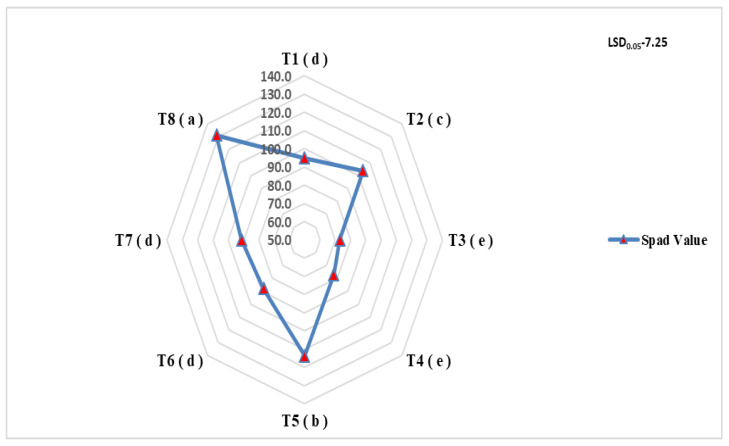
Impact of organic manures on leaf Spad values for *Actinidia deliciosa* cv ‘Allison’.

A perusal of data presented in Figure 1 revealed that different treatments of organic manures also exhibited a significant influence on leaf chlorophyll content. The vines treated with the recommended dose of nutrients exhibited the highest spad value, which was followed by vine-treated RDN through DM and VC. However, the lowest chlorophyll content was recorded in vines treated with 100 % poultry manure.

**Figure 2:**
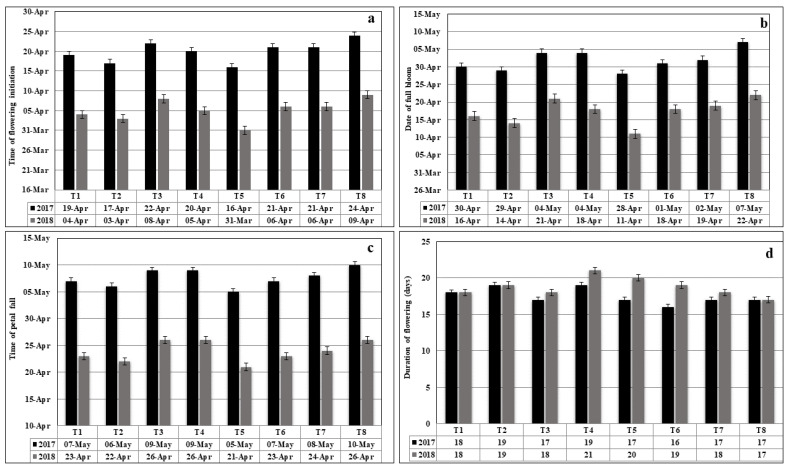
Impact of organic manures on (**a**) time of flower initiation (**b**) date of full bloom (**c**) time of petal fall; (**d**) duration of flowering of *Actinidia deliciosa* cv ‘Allison’.

The data about the effect of organic manure on the blooming characteristics revealed that the organic manure applications exhibited significant influence on: the time of flower initiation; date of full bloom; time of petal fall; and duration of flowering of *Actinidia deliciosa* cv ‘Allison’. The advances in vines treated with different organic manures are presented in Figure 2a–d. The maximum advancement was noticed in treatment DM coupled with VC (50:50) as compared to other treatments. The organic manure applications had a substantial effect on the date of full bloom and followed a more or less similar trend as that of flower initiation. The earliest date of full bloom was observed when DM and VC treatment were applied in equal proportions. Similarly, the data on the date of petal fall was advanced because of the application of the different organic manures. During both years, the vines treated with DM and VC (50:50) resulted in the earliest petal fall, which was reported on 4 May and 21 April 2017 and 2018, respectively. This treatment advanced the petal fall by 5 days and was closely followed by the vines treated with VC (100 %), which resulted in the petal fall on 6 May and 22 May during 2017 and 2018, respectively. This treatment enhanced the petal fall by 4 days in comparison to the control. The data about the duration of the flowering period indicated that different organic manures observed the highest duration of flowering, i.e., 19 and 21 days during treatment with DM and chicken manure (50:50) during 2017 and 2018, respectively (Figure 2). The lowest flowering period of 17 days was recorded in NPK + 40 kg DM during both years.

### 2.2. Growth Characteristics

The number of leaves per plant, total leaf area per plant, and stem diameter increment in the kiwi fruit plant changed and exhibited substantial impacts with the application of different organic manures (Table 1). The number of leaves per plant ranged from 298 to 408, and the NPK with 40 kg DM had substantially more than the other organic treatments. The average leaf area per plant followed a remarkably similar trend. The NPK +40 kg DM, or recommended fertilizer dosage, had the highest total leaf area. The NPK +40 kg DM exhibited the greatest increase in stem diameter.

### 2.3. Yield and SSC

The fruit production estimates shown in Table 2 are based on the average of two years of nutrient treatments. The impact of organic manure applications on fruit yield per vine differed considerably between treatments. The highest fruit yield (24.86 kg vine^−1^) was noticed with the NPK + 40 kg DM followed by DM and VC (50:50), recording 18.17 kg vine^−1.^ This treatment produced the highest fruit yield amongst all organic input treatments other than the control (T8). The lowest fruit yield (12.23 kg vine^−1^) was obtained with chicken manure (100%), which was statistically almost similar to the yield obtained through the application of DM, CM, and VC in equal proportions and DM and chicken manure (50:50), recording 13.68 and 13.52 kg fruit per vine, respectively. In terms of the fruit quality parameters such as soluble solid content at the time of harvest, the highest SSC, i.e., 7.64 %, was recorded with treatment vermicompost (100%), which was similar to that recorded with DM and VC (50:50), recording 7.46 %.

### 2.4. Leaf Nutrient Status

The macro- and micronutrient uptakes, namely, N, P, K, Fe, Zn, Cu, and Mn in both harvests showed a substantial effect. Treatment in VC coupled with DM manures brought about the highest nutrient uptake in the leaves of kiwifruit (Table 3). The data showed that leaf nitrogen content significantly varied from 1.57 to 2.57 %. The highest leaf nitrogen content (2.57%), phosphorus content (0.38%), and potassium (1.81%) were noticed in the fruits produced through the combined application of vermicompost and DM (50:50). Micronutrient contents of the leaf were also influenced significantly by different OM treatments in an almost similar trend. Leaf Fe, Cu, Zn, and Mn contents significantly varied from 183.01 to 210.00, 14.68 to 20.22, 33.87 to 38.53, and 61.22 to 78.39 mg/kg, respectively, amongst the treatments. The highest iron (210 ppm), copper (20.22), zinc (38.53), and manganese (78.39) content were also recorded with the application of vermicompost and DM in equal proportions.

### 2.5. Physico-Chemical Properties of Soil

The application of different OM treatments had a substantial effect on the physical properties of soil (Table 4). However, the highest pH in soil was recorded with treatments involving vermicompost (100%), DM and Vermicompost (50:50), and CM and vermicompost (50:50) with similar soil pH, whereas the lowest soil pH (6.73) was recorded in DM, CM, and VC (equal proportion), which was statistically at par with T_1_ = T_3_ and T_8_ similar soil pH values of 6.74. Organic manures showed a substantial effect on organic carbon as affected by different organic manure applications, with values ranging from 0.87 to 1.05 %. The highest organic carbon concentration (1.05%) was found in DM and vermicompost (50:50), which was statistically equivalent to vermicompost (100%) at 1.04 % organic carbon content. OM showed significant differences under the greatest water-holding capacity of the soil (MWHC). The highest MHWC (35.79 %) was attained with the treatment of vermicompost (100%), which was statistically similar to that obtained by T_5_ > T_1_ and T_4_ (Table 5). The different organic manure treatments had a substantial influence on the cation exchange capacity (CEC) of soil ranging from 11.78 to 14.01 meq/100 g. The highest CEC of soil (14.01 meq/100 g) was reported with DM and VC (50:50), which was found statistically equivalent to that obtained through vermicompost (100%) and DM (100%), recording 13.87 and 13.81 meq/100 g, respectively. Different OM treatments revealed a wide range (254.33 to 299.03 kg/ha) of available N and indicated that different organic treatments exerted a marked influence on the available N in the soil. The highest available N was noticed with (NPK + 40 kg DM) which was statistically similar to that with chicken manure (100%), recording 292.0 kg/ha available N in the soil. Even though the different treatments exhibited remarkable differences in the available P content in soil, they followed an almost similar trend as followed by N. It is evident from the data that the highest available phosphorus (52.63 kg/ha) was observed under NPK + 40 kg DM, which was found statistically similar to that obtained through the application of manure (100 %), recording 52.11 kg/ha available phosphorus. However, the lowest available phosphorus (36.1 kg/ha) was recorded with DM (100%).

## 3. Discussions

Several studies have demonstrated that increasing food availability has a positive impact on chlorophyll content [18,19,20]. This positive effect was also found when N was supplied, particularly in the application of organic manures coupled with NPK, corroborating Akram’s findings [19]. Vermicompost and DM may have a positive influence on spad values, since this influence and change numerous enzymatic activities in plants that promote cell elongation, root and shoot development, and glucose metabolism. The findings of Karibasappa et al. [21] and Baksh et al. [22] have substantiated these findings. The current findings revealed that applying farmyard manure and vermicompost separately had a significant influence on blooming and fruiting. Early flowering and a larger number of flowers after applying DM and vermicompost might be attributed to increased nitrogen and phosphorus levels. They were shown to be more effective in synthesizing growth promoters and producing photosynthates. These results are consistent with those of Arancon et al. [23] in strawberries and Yadav et al. [24] in papaya. Since certain organic manures are nutrient deficient, the experiments with ‘Allison’ kiwifruit revealed that increasing leaf area with suitable fertilizer dosages together with dairy manures results in a substantially larger leaf area than organic manures.

However, Nitrogen and Phosphorus fertilizers led to an increase in the total leaf area, because fertilizer application mainly increases the size of leaves, whereas phosphorus fertilizer mainly increases the number of leaves. A similar outcome has been confirmed for *Picea sitchensis*, with increased leaf area caused by increased average cell size, more cells per leaf, and a quicker rate of cell growth due to increased nitrogen availability [25]. The increase in stem diameter was typically followed by canopy reactions, fertilizer application experiments, and fruit crop trials, in that order [26]. During the investigation, total leaf area and stem diameter increased substantially in response to fertilizer application, with NPK + 40 kg DM providing the best results. With the application of the NPK + 40 kg DM, the growth rates may be linked to improvements in photosynthetic rate and total leaf area, resulting in increased yield. In contrast to inorganic manures, organic manures have a restricted supply of nutrients, which causes the plant to have inappropriate vegetative development. With the application of the NPK + 40 kg DM, the growth rates may be linked to improvements in photosynthetic rate and total leaf area, resulting in increased yield.

The increase in yield with application of NPK and DM that improves the nutrients, moisture, and growth-promoting chemicals boosts the plant’s metabolic and hormonal activity, allowing more photosynthates to be stored as starch and carbohydrates in the fruits [27]. The optimal nutrition supply improved fruit output as well. In perennial fruit crops, greater fruit yield and quality have been reported after manure and fertilizer application [28]. Inorganic sources of nutrients increased the production of kiwifruit and pineapple, according to Guarconi and Ventura [29]. These findings corroborate those of Garg et al. [17] and Yadav et al. [24]. Organic manure applications had a substantial impact on the soluble solid contents in terms of quality (SSC). Organic manures are hydrophilic, which causes them to absorb moisture and nutrients for a longer period in the soil, thereby improving soil structure and indirectly improving fruit quality. Organic manures, particularly vermicompost and DM contribute to a greater C/N ratio and critical plant nutrients necessary for improved metabolic activities in the plant, increasing protein and carbohydrate synthesis and, as a result, increasing fruit production [30]. Similar results have been obtained by Odongo et al. [31], who discovered that dairy manures substantially reduced the soluble solid content [32]. Korwar et al. [33] found that when inorganic fertilizers and OM were used together, the yield of aonla was higher. Similarly, the increased availability of macro and micronutrients in the leaves of kiwifruit vines caused by adding vermicompost and dairy manures improves the physical condition of the soil and root development by increasing moisture retention, thus increasing water and nutrient absorption. Microorganisms’ activities are enhanced with the application of organic manures. Hence, N absorption increases and changes organically bound N into an inorganic form in plants [9].

The higher phosphorus content observed with DM and VC might be ascribed to increased P availability in the rhizosphere. The multifaceted organic anions chelate Al^+3^, Fe^+3^, and Ca^+2^ and reduce these cations’ phosphate precipitating power, increasing phosphorus availability. Moreover, organic residues can cause plants to generate oxalic acid, which results in the mobilization of immobile phosphorus and aids in the increase in phosphorus absorption by plants. Organics increased micronutrient uptake in various fruit crops, including guava, pomegranate, and kiwifruit [17]. The contents of the leaf were also significantly influenced by different organic treatments. Soil biological activity was enhanced by the addition of OM, which aids in the solubilization of nutrients, their availability to plants, and their absorption. According to Garg et al. [17], organic manures, alone or in conjunction with inorganic fertilizers, enhanced the availability and absorption of Fe, Zn, Cu, and Mn in sweet oranges. The present investigation revealed that organic manure enhances the physical parameters better than the chemical properties of the soil. Soil pH has been linked to a boost due to its ability to control soil fertility. This could be attributed to the addition of organic matter to the soil and proton release, resulting in the buildup of organic anions in plants, viz., malate, citrate, and oxalate. Similarly, considerable organic matter inputs in kiwifruit have been attributed to higher organic carbon in organic systems than in conventional agricultural methods.

Organic manure decomposition enhances the organic matter constitution of the soil, increasing the soil’s organic carbon. The findings of the present study are consistent with the observations of Kumari et al. [34] and Marzi et al. [35]. The addition of organic manure increases the organic matter and pH of the soil. Continuously applying organic manures may result in a decrease in bulk density and a corresponding increase in the soil’s maximum water-holding capacity. Organic elements enhanced the surface soil’s organic carbon, aggregate stability, moisture retention capacity, and infiltration rate while lowering the bulk density. The findings of the present study are consistent with those of Garg et al. [17] and Manjunatha et al. [36].

The rise in the available nitrogen content caused by the addition of inorganic fertilizers and organic manures might be ascribed to increased soil microbial proliferation. During mineralization, it changes nitrogen that is bound to organic molecules into nitrogen that is bound to inorganic molecules. This makes nitrogen in the soil easier to access. Different sources of nitrogen and organic carbon, as well as organic manures, are used to build up nitrogen and organic carbon in the soil. The maximum available N in soil with the application of DM and inorganic fertilizers was also noticed by Venugopal and Sheela [37] in bananas. The highest available nitrogen content in soil treatment T_8_ (NPK+ 40 kg DM) may be attributed to the residual effect of inorganic nitrogen fertilizers. Organic acids formed during the breakdown of organic manures react to convert the unavailable P in the soil into readily available forms. The decomposition of native soil organic matter (SOM) is accelerated, resulting in increased mineralization and nutrient release. The results of this study back up the results of Marimuthu et al. [38], who found that different amounts of organic manures and inorganic fertilizers increased the NPK content of the soil.

## 4. Materials and Methods

### 4.1. Study Area and Plant Materials

From 2016 to 2018, the field trial was conducted in an experimental block of the Department of Fruit Science, Dr. YSP University of Horticulture and Forestry, Nauni, Solan (HP), at an elevation of 1260 m above mean sea level, with a latitude of 30°50′18″ N and a longitude of 77°11′30″ E. The experimental field is in sub-temperate, sub-humid, and mid-hill agro-climatic zones in the state of Himachal Pradesh (Zone-II). The study area’s average annual rainfall is around 100–130 cm, with most of it falling between July and September. In the plowed layer (0 to 20 cm), the soil texture was found to be sandy loam. Kiwifruit vines of the variety ‘Allison’ were carefully chosen for the experiment and were planted in 2009, T-bar trained, with rows oriented north–south at a spacing of 4.0 m × 6.0 m (416 vines ha^−1^), the female: male ratio was 9:1. Before the start of the experiment, the physico-chemical characteristics of orchard soil were recorded as the following: pH 6.75, the electrical conductivity of 0.15 dS m^−1^, and organic carbon of 0.82%. Surface soil had 250.55, 40.00, and 260.35 kg/ha of available N, P, and K, respectively. The climate of the experimental area is characterized by sub-tropical in the valley which is renowned as the mid-hills of the Himalayas. It is a bracing hill station throughout the year but hotter as compared to Shimla. Figure S1 shows the mean weekly weather parameters for both years 2017 and 2018 during the experimentation period that was recorded at the Meteorological observatory. The average annual rainfall in this area was about 120–130 cm, out of which 85% of rainfall is received from June to September.

### 4.2. Experimental Design

The experiment involved 8 treatments: (1) T_1_: 100% Dairy manure (DM); (2)T_2_: 100% Vermicompost (VC); (3) T_3_: 100% chicken manure (CM); (4) T_4_: 50% DM + 50% CM; (5) T_5_: 50% DM + 50% VC; (6) T_6_: 50% CM + 50% VC; (7) T_7_: DM + CM + VC in equal proportions; and (8) T_8_: Recommended nutrients inorganic NPK + 40 kg DM. The experiment was carried out using a randomized complete block design with three replicas. The two common applications of neem @1kg per vine and spray of the liquid formulation after fruit set except T_8_ were also applied.

### 4.3. Nutrient Application

The mineral fertilizers such as urea; 46% N, Super phosphate; 16% P, and potassium chloride; 60% were used as sources of N, P, and K, respectively. The composted manure based on testing contained: dairy manure-0.5% N: 0.25% P: 0.5% K; VC-3.05% N: 2.0% P: 0.5% K; and CM-2.05% N: 1.5% P: 1.0% K. Organic material was incorporated thoroughly into the respective plots four weeks after the dormancy period. Chemical fertilizers were applied during each growing season. The N was employed in two split doses; the first half dose was applied during February, while the second half was applied about two weeks before flowering. Full doses of P and K were applied with the first split dose of nitrogen during February. Mineral fertilizers were mixed and then applied in the basin of the vine, a system generally used by the local farmer community. The source of irrigation was a drip irrigation. The calculations of the applied nutrients have been presented in Table 5.

### 4.4. SPAD Readings and Flowering Characteristics

A portable chlorophyll meter was used for gauging the total chlorophyll content. (SPAD 502 plus chlorophyll meter, Konica Minolta, Osaka, Japan) on 25 leaf samples per treatment. Three relative readings per leaf were averaged to denote one observation. SPAD readings showed a positive correlation with leaf N and chlorophyll concentration [39] and were estimated as SPAD units. The date of flower opening on the shoots was recorded and expressed as the time of flower initiation. The date on which more than 75% of flowers opened on the vine was noted and reckoned as the date of full bloom, and the date of petal fall, when all petals fell after full fruit setting, was recorded, and expressed as the time of petal fall; in contrast, the duration of flowering was recorded from the initiation of flowering to petal fall and expressed as a number of days.

### 4.5. Growth Measurements

On 20 December 2016, before the treatments were performed, the ground-level base diameter of the vine was measured. The final seedling size was measured in October 2017 and 2018. Growth increment is the difference between the final and initial size of the trunk. Basal diameter (trunk girth) was measured using a Vernier caliper. Ten fully expanded leaves from each experimental tree were randomly collected in June. The leaf area was recorded with the LI-Cor-3000 C Portable Leaf Area Meter (Lincoln, NE, USA), while the values were stated as average leaf area per leaf in square centimeters (cm^2^). One year later, adding fertilizer changed the number of leaves per plant, the total leaf area per plant, the diameter of the new shoot, and the growth of the stem diameter.

### 4.6. Fruit Sampling and Analysis

Fruits were harvested on the 6th and 18th of October in 2017 and 2018, respectively, when the soluble solid contents reached a minimum of 6.2° Brix. The pre-harvest soluble solids content (SSC) was measured weekly (from the first week of October onwards). The harvested fruits were also utilized for analyzing physical-biochemical characteristics using standard procedures [40]. The SSC was determined with the help of a Digital refractometer (0–32° Brix).

### 4.7. Leaf Sampling and N, P, K Determination

An illustrative leaf sample of 50 completely matured and extended current season leaves placed at the 8th to 10th position from the apex was collected procedures [41]. The leaf samples thus collected were thoroughly washed sequentially with water, aqueous soap, acidic water, and glass re-distilled water. The leaves were shade dried for four days before being dried in the oven, at 65–70 °C, until they attained a persistent weight. The dried samples were then crushed and ground into a powder, mixed well, and a micro-Kjeldahl distillation technique was employed to calculate the total nitrogen, which was digested with a Tri-acid combination. The samples were then digested in HNO_3_-HClO_4_ at a 9:4 *v*/*v* ratio (Di-acid mixture), which was applied for analyzing P using the vanadomolybdophosphoric acid method, K by using a flame photometer, Calcium, and magnesium with a titrimetric method using disodium salt of EDTA procedures [42]. An atomic absorption spectrophotometer (AAS-Perkin Elmer, NJ, USA Analyst 400, Waltham, USA) was used to examine micronutrients such as zinc, manganese, iron, and copper.

### 4.8. Physicochemical Properties of Soil

The soil samples from 0–45 cm depth were collected before the start of the experiment and at the end of the experiment. Before the start of the experiment a composite sample of the orchard soil was collected, whereas at the end of the experiment, samples were collected from four sides under the basin of each experimental tree with the help of a screw-type auger. The soil samples, thus collected were dried in shade, grounded, sieved through a 2 mm plastic sieve, and stored in cloth bags. The soil pH was calculated in a 1:2.5 (*w*/*v*) aqueous solution using a 1:2.5 fresh soil–water suspension with ideal pH. The organic carbon (OC) content of dry soil samples was measured by Wakley and Black’s method [43]. The keen and Raczkowski box technique (1966), as described by Piper procedures [44], was used to measure the maximum water holding capacity (WHC).

The alkaline potassium permanganate technique, as defined by Subbiah and Asija procedures [45], was used to calculate nitrogen (N), which was expressed in kg/ha. Olsen’s extractant procedures [46] were used to quantify phosphorus in soil and were determined on a UV-Spectrophotometer at 660 nm wavelength. Available phosphorus (P) was estimated by Olsen’s extractant (0.05 N NaHCO_3_, pH 8.50) method procedures [47]. The same was estimated on a flame photometer and expressed in kilograms per hectare. The method determined the average soil’s cation exchange capacity (CEC) as described by Bower et al. [48] procedures. Sodium concentration was estimated by a flame-photometer using a standard series of NaCl.

### 4.9. Statistical Analysis

The data analyses were carried out using SPSS-21 software (SPSS, Chicago, IL, USA). The significance of differences between treatments for the different measured parameters was evaluated by one-way ANOVA followed by Duncan’s multiple range test (*p* < 0.05).

## 5. Conclusions

The results of this study show that using organic and inorganic fertilizers has unique advantages. The use of inorganic fertilizers (NPK) in combination with DM enhanced spad values chlorophyll, fruit production, leaf number, leaf area, and stem diameter while also improving the soil’s chemical characteristics. The flower initiation were recorded with DM and Vermicompost (50:50). Furthermore, when compared to inorganic fertilizer treatment, OM treatment significantly improved fruit quality by improving fruit chemical composition in terms of soluble solids contents and leaf nutrient status, as well as improving soil’s physical properties with DM and Vermicompost (50:50). The study’s outcome revealed that OM had a significant impact on flowering time, leaf nutritional status, and soil physical characteristics. In comparison to organic treatments, recommended fertilizer dosages (NPK+DM) improved plant growth, fruit yield, and soil chemical characteristics. The combined organic manures and inorganic fertilizer application, i.e., NPK +40 kg DM, suggested that the fertilizer dose could promote the growth and fruit yield of ‘Allison’ kiwifruit. The consolidated application of organic manures improves the SSC and nutrient uptake of the kiwifruit.

## Figures and Tables

**Table 1:** Impact of organic manures on the plant growth characteristics of *Actinidia deliciosa* cv ‘Allison’.

Code	Treatments	Total no. of Leaves	Total Area of Leaves per Plant (m^2^)	Stem Diameter Increase
T_1_	100% Dairy manure (DM)	328 ± 4.16 ^d^	3.81 ± 0.09 ^d^	0.32 ± 0.001 ^d^
T_2_	100% Vermicompost (VC)	349 ± 8.33 ^c^	4.09 ± 0.07 ^c^	0.36 ± 0.01 ^c^
T_3_	100% Chicken manure (CM)	298 ± 6.81 ^f^	3.33 ± 0.07 ^f^	0.22 ± 0.01 ^g^
T_4_	50% DM+ 50% CM	303 ± 6.24 ^ef^	3.40 ± 0.01 ^f^	0.27 ± 0.01 ^f^
T_5_	50% DM + 50% VC	370 ± 0.88 ^b^	4.30 ± 0.01 ^b^	0.39 ± 0.01 ^b^
T_6_	50% CM + 50% VC	313 ± 4.16 ^def^	3.63 ± 0.04 ^e^	0.29 ± 0.01 ^e^
T_7_	DM + CM + VC in equal proportions	315 ± 4.13 ^de^	3.64 ± 0.01 ^e^	0.30 ± 0.01 ^e^
T_8_	Recommended dose of fertilizers	408 ± 9.89 ^a^	4.83 ± 0.12 ^a^	0.46 ± 0.01 ^a^
	* LSD (*p* 0.05)	18.09	0.20	0.01

* LSD = Least Significance difference; the values shown are the mean ± SE. Different letters in the same column indicate a significant difference at the *p* = 0.05 level.

**Table 2:** Response of nutrient supplementation through organic manures on the yield and soluble solids content of *Actinidia deliciosa* cv ‘Allison’.

Code	Treatments	Yield (kg plant^−1^)	Soluble Solid Contents (%)
T_1_	100% Dairy manure (DM)	16.62 ± 0.02 ^d^	7.12 ± 0.54 ^b^
T_2_	100% Vermicompost (VC)	17.23 ± 0.19 ^c^	7.64 ± 0.12 ^a^
T_3_	100% Chicken manure (CM)	12.23 ± 0.12 ^f^	6.63 ± 0.10 ^d^
T_4_	50% DM+ 50% CM	13.52 ± 0.05 ^e^	6.64 ± 0.06 ^d^
T_5_	50% DM + 50% VC	18.17 ± 0.02 ^b^	7.46 ±0.07 ^a^
T_6_	50% CM + 50% VC	15.23 ± 0.38 ^d^	6.86 ± 0.11 ^c^
T_7_	DM + CM + VC in equal proportions	13.68 ± 0.32 ^e^	6.79 ± 0.05 ^cd^
T_8_	Recommended dose of fertilizers	24.86 ± 0.31 ^a^	6.33 ± 0.06 ^e^
	* LSD_0.05_	2.43	0.46

* LSD = Least Significance difference; the values shown are the mean ± SE. Different letters in the same column indicate a significant difference at the *p* = 0.05 level.

**Table 3:** Impact of organic manures on leaf nutrients status of *Actinidia deliciosa* cv ‘Allison’.

Code	Treatments	Macro Nutrients (%)			Trace or Micronutrients Elements (mg kg^−1^)			
		N	P	K	Fe	Cu	Zn	Mn
T_1_	100% Dairy manure (DM)	2.47 ± 0.03 ^a^	0.24 ± 0.01 ^c^	1.69 ± 0.03 ^bc^	201.24 ± 4.50 ^abc^	18.02 ± 0.28 ^c^	35.97 ± 0.098 ^bc^	69.99 ± 1.52 ^c^
T_2_	100% Vermicompost (VC)	2.50 ± 0.01 ^a^	0.32 ± 0.01 ^b^	1.75 ± 0.04 ^ab^	206.45 ± 0.75 ^ab^	19.09 ± 0.31 ^b^	36.73 ± 0.12 ^ab^	74.22 ± 1.08 ^b^
T_3_	100% Chicken manure (CM)	1.90± 0.01 ^d^	0.18 ± 0.001 ^e^	1.34 ± 0.02 ^e^	187.88 ± 4.69 ^de^	14.97 ± 0.11 ^fg^	34.00 ± 0.77 ^d^	63.31 ± 0.63 ^d^
T_4_	50% DM+ 50% CM	2.07 ± 0.03 ^c^	0.21 ± 0.01 ^d^	1.45 ± 0.04 ^d^	196.10 ± 4.38 ^cd^	15.78 ± 0.08 ^de^	34.20 ± 0.83 ^cd^	64.33 ± 0.97 ^d^
T_5_	50% DM + 50% VC	2.57 ± 0.03 ^a^	0.38 ± 0.01 ^a^	1.81 ± 0.01 ^a^	210.00 ± 0.87 ^a^	20.22 ± 0.15 ^a^	38.53 ± 0.95 ^a^	78.39 ± 1.51 ^a^
T_6_	50% CM + 50% VC	2.20 ±0.01 ^b^	0.19 ± 0.01 ^e^	1.487 ± 0.02 ^d^	192.33 ± 0.60 ^cde^	15.33 ± 0.24 ^ef^	34.57 ± 0.74 ^cd^	68.42 ± 1.67 ^c^
T_7_	DM + CM + VC in equal proportions	2.27 ± 0.03 ^b^	0.23 ± 0.01 ^c^	1.64 ± 0.04 ^c^	199.50 ± 3.28 ^bc^	16.05 ± 0.12 ^d^	35.47 ± 0.30 ^bcd^	69.12 ± 1.27 ^c^
T_8_	Recommended dose of fertilizers	1.57 ± 0.03 ^e^	0.16 ± 0.01 ^f^	1.31 ± 0.01 ^e^	183.01 ± 0.28 ^e^	14.68 ± 0.13 ^g^	33.87 ± 0.33 ^d^	61.22 ± 0.31 ^d^
	* LSD_0.05_	0.11	0.02	0.09	9.90	0.57	1.96	3.88

* LSD = Least Significance difference; the values shown are the mean ± SE. Different letters in the same column indicate a significant difference at the *p* = 0.05 level.

**Table 4:** Impact of organic manures on physicochemical properties of soil in kiwifruit orchard.

Code	Treatments	pH	OC%	CEC Meq/100 g	MWHC (%)	Avail. N	Avail. P	Avail. K
T_1_	100% Dairy manure (DM)	6.74 ± 0.04 ^b^	1.02 ± 0.01 ^abc^	13.82 ± 0.17 ^abc^	35.08 ± 0.88 ^ab^	254.33 ± 4.22 ^d^	36.10 ± 0.09 ^f^	247.44 ± 3.78 ^bc^
T_2_	100% Vermicompost (VC)	6.78 ± 0.02 ^a^	1.04 ± 0.01 ^ab^	13.87 ± 0.03 ^abc^	35.79 ± 0.63 ^a^	264.33 ± 3.57 ^c^	48.25 ± 1.02 ^b^	262.67 ± 5.05 ^ab^
T_3_	100% Chicken manure (CM)	6.74 ± 0.06 ^b^	0.94 ± 0.01 ^e^	12.89 ± 0.13 ^d^	33.70 ± 0.4 ^b^	292.00 ± 3.191 ^a^	52.13 ± 0.46 ^a^	245.59 ± 9.13 ^c^
T_4_	50% DM + 50% CM	6.75 ± 0.01 ^b^	0.96 ± 0.01 ^de^	13.30 ± 0.11 ^cd^	34.58 ± 0.38 ^ab^	260.93 ± 3.26 ^cd^	37.66 ± 0.02 ^e^	248.06 ± 4.77 ^bc^
T_5_	50% DM + 50% VC	6.78 ± 0.09 ^a^	1.05 ± 0.01 ^a^	14.01 ± 0.11 ^a^	35.12 ± 0.58 ^ab^	260.10 ± 0.95 ^cd^	41.16 ± 0.38 ^c^	260.07 ± 3.38 ^bc^
T_6_	50% CM + 50% VC	6.78 ± 0.16 ^a^	1.00 ± 0.01 ^bcd^	13.59 ± 0.29 ^abc^	33.55 ± 0.50 ^b^	277.10 ± 3.75 ^b^	39.70 ± 0.12 ^d^	256.40 ± 0.66 ^bc^
T_7_	DM + CM + VC in equal proportions	6.73 ± 0.07 ^c^	0.99 ± 0.01 ^cde^	13.39 ± 0.19 ^bcd^	33.40 ± 71 ^b^	267.33 ± 1.53 ^c^	38.03 ± 0.09 ^e^	252.00 ± 6.29 ^bc^
T_8_	Recommended dose of fertilizers	6.74 ± 0.04 ^b^	0.87 ± 0.01 ^e^	11.78 ± 0.29 ^e^	30.83 ± 0.45 ^c^	299.03 ± 0.15 ^a^	52.63 ± 0.05 ^a^	276.29 ± 1.86 ^a^
* LSD_0.05_		** NS	0.05	0.33	1.84	7.97	3.86	3.76

OC% = Organic carbon %age; CEC = Cation exchange capacity; MHWC = Maximum water holding capacity. * LSD = Least Significance difference; the values shown are the mean ± SE. Different letters in the same column indicate a significant difference at the *p* = 0.05 level. ** NS = Non-significant.

**Table 5:** Nutrient application rates for Kiwifruit orchards during the experiment.

Code	Treatment	Quantity (kg vine^−1^)	Kiwifruit Orchards g vine^−1^		
			N	P	K
T_1_	100% Dairy manure	200.00 *	1000	500	1000
T_2_	100% VC	48.80 *	1000	732	488
T_3_	100% CM	32.80 *	1000	656	492
T_4_	50% Dairy manure + 50% CM	100.00+ 16.40 *	500 + 500	225 + 328	500 + 246
T_5_	50% Dairy manure + 50% VC	100.00 + 24.4 *	500 + 500	225 + 366	500 + 244
T_6_	50% CM + 50% VC	16.50 + 24.4 *	500 + 500	328 + 366	246 + 244
T_7_	Dairy manure + CM + VC in equal proportions	66.50 + 10.93 +16.24 *	333.3 + 333.3 + 333.3	166.7 + 244.0 + 218.7	333.3 + 162.7 + 164
T_8_	Recommended dose of fertilizers	FYM-40.00 + urea-1.74 + SSP-3.75 + MOP-1.33	200 + 800 g	100 + 600 g	200 + 800 g

* Dose is calculated based on nutrient content as well as N equivalence of Recommended dose of fertilizer (FYM 40 kg+ 800 N- 600 P:800 K). VC—Vermicompost; FYM—Farmyard manure, MOP—Muriate of Potash; SSP—Single super Phosphate; CM—Chicken Manure; Poultry manure.

## Data Availability

Raw data is available on request declaration.

## Supplementary Materials

The following supporting information can be downloaded at: www.mdpi.com/article/10.3390/plants11233354/s1, Figure S1: Meteorological observation of the experimental orchard.
