# Supplementary figures and images for: Effect of Organic Manures on Growth, Yield, Leaf Nutrient Uptake and Soil Properties of Kiwifruit (Actinidia deliciosa Chev.) cv. Allison
Source: Plants (Basel). 2022 Dec 2;11(23):3354. doi: 10.3390/plants11233354 (PMC9741236; doi:10.3390/plants11233354)

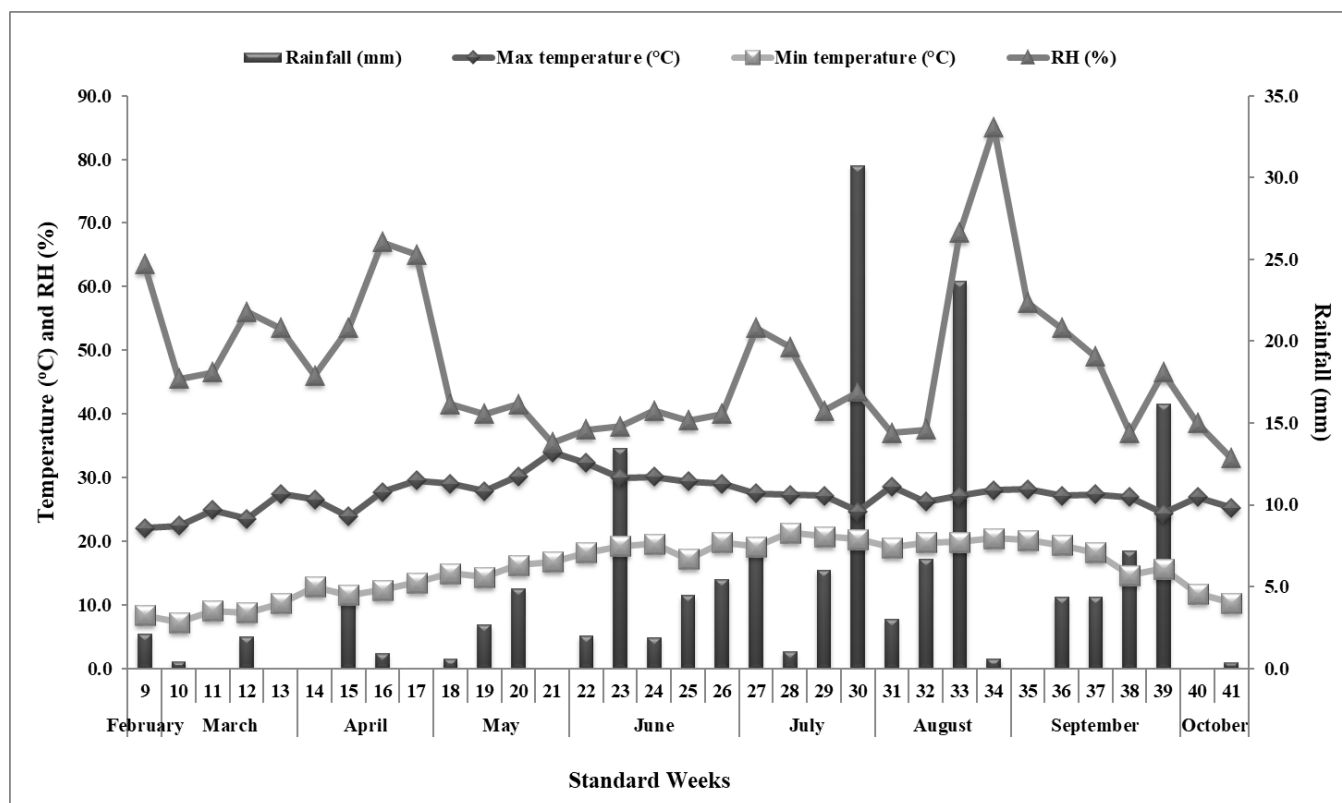

Figure S1. Meteorological observation of the experimental orchard.

Supplement: Supplementary file 1 [file plants-11-03354-s001.zip › plants-2042185-supplementary.pdf]
